# Syncytiotrophoblast extracellular vesicles impair rat uterine vascular function via the lectin-like oxidized LDL receptor-1

**DOI:** 10.1371/journal.pone.0180364

**Published:** 2017-07-03

**Authors:** Floor Spaans, Cindy K. Kao, Jude S. Morton, Anita L. Quon, Tatsuya Sawamura, Dionne S. Tannetta, Ian L. Sargent, Sandra T. Davidge

**Affiliations:** 1 Department of Obstetrics and Gynecology, University of Alberta, Edmonton, AB, Canada; 2 Women and Children's Health Research Institute, University of Alberta, Edmonton, AB, Canada; 3 Department of Physiology, Shinshu University School of Medicine, Asahi, Matsumoto, Japan; 4 Nuffield Department of Obstetrics & Gynaecology, University of Oxford, John Radcliffe Hospital, Headington, Oxford, United Kingdom; 5 Department of Physiology, University of Alberta, Edmonton, AB, Canada; University of Southampton, UNITED KINGDOM

## Abstract

Syncytiotrophoblast extracellular vesicles (STBEVs) are placenta derived particles that are released into the maternal circulation during pregnancy. Abnormal levels of STBEVs have been proposed to affect maternal vascular function. The lectin-like oxidized low-density lipoprotein receptor-1 (LOX-1) is a multi-ligand scavenger receptor. Increased LOX-1 expression and activation has been proposed to contribute to endothelial dysfunction. As LOX-1 has various ligands, we hypothesized that, being essentially packages of lipoproteins, STBEVs are able to activate the LOX-1 receptor thereby impairing vascular function via the production of superoxide and decreased nitric oxide bioavailability. Uterine arteries were obtained in late gestation from Sprague-Dawley rats and incubated for 24h with or without human STBEVs (derived from a normal pregnant placenta) in the absence or presence of a LOX-1 blocking antibody. Vascular function was assessed using wire myography. Endothelium-dependent maximal vasodilation to methylcholine was impaired by STBEVs (MCh E_max_: 57.7±5.9% in STBEV-incubated arteries vs. 77.8±2.9% in controls, p<0.05). This was prevented by co-incubation of STBEV-incubated arteries with LOX-1 blocking antibodies (MCh E_max_: 78.8±4.3%, p<0.05). Pre-incubation of the vessels with a nitric oxide synthase inhibitor (L-NAME) demonstrated that the STBEV-induced impairment in vasodilation was due to decreased nitric oxide contribution (ΔAUC 12.2±11.7 in STBEV-arteries vs. 86.5±20 in controls, p<0.05), which was abolished by LOX-1 blocking antibody (ΔAUC 98.9±17, p<0.05). In STBEV-incubated vessels, LOX-1 inhibition resulted in an increased endothelial nitric oxide synthase expression (p<0.05), to a level similar to control vessels. The oxidant scavenger, superoxide dismutase, did not improve this impairment, nor were vascular superoxide levels altered. Our data support an important role for STBEVs in impairment of vascular function via activation of LOX-1 and reduced nitric oxide mediated vasodilation. Moreover, we postulate that the LOX-1 pathway could be a potential therapeutic target in pathologies associated with vascular dysfunction during pregnancy.

## Introduction

STBEVs are heterogeneous membranous vesicles released into the maternal circulation by the multinucleated syncytiotrophoblast layer of the placenta. They are variable in size, ranging from smaller exosomes and ectosomes (50–150 nm) to larger extracellular vesicles (100 nm—1 μm) [1], and during pregnancy they are present from the second trimester onwards, reaching their highest levels at the end of gestation [2, 3]. While STBEVs are naturally released during pregnancy, increased concentrations of STBEVs have been suggested to contribute to potential pathological states such as systemic inflammation and endothelial dysfunction [4]. Specifically, STBEVs have been shown to activate peripheral blood monocytes and peripheral blood mononuclear cells [2, 5], disrupt the monolayer architecture and reduce proliferation of endothelial cells [6]. In addition, some *ex vivo* studies have shown that STBEVs affect vascular function [7] while others did not observe any changes [8]. Although it has been suggested that the proteins and the danger-associated molecular patterns (DAMPs) that STBEVs carry on their surface (such as heat shock proteins) could mediate their own activity [1], the specific receptor(s) by which STBEVs act and their exact mechanism(s) of downstream action are still unknown.

The lectin-like oxidized low-density lipoprotein receptor-1 (LOX-1) is the main receptor involved in the uptake of oxidized low-density lipoprotein (oxLDL) and it has been well-studied in cardiovascular diseases such as atherosclerosis [9] and has been shown to be increased in preeclampsia [10–12], which is characterized by systemic endothelial dysfunction. Activation of LOX-1 by oxLDL impairs vascular function [13] via increased NADPH oxidase activation, and superoxide and peroxynitrite production [12]; leading to decreased nitric oxide (NO) bioavailability [14–16]. In addition, oxLDL stimulation was shown to reduce endothelial nitric oxide synthase (eNOS) expression in endothelial cells *in vitro*, which was LOX-1 dependent [17, 18]. Further, previous studies from our group have shown that increased LOX-1 expression may be implicated in impaired endothelium-dependent vasodilation during pregnancy [12]. Moreover, in pregnant animals with reduced uterine perfusion pressure aortic LOX-1 expression was increased and was suggested to play a role in the observed endothelial dysfunction [11].

In addition to oxLDL, many other factors have been shown to be ligands for LOX-1 such as: other modified lipoproteins, activated platelets, apoptotic cells and even bacteria [19, 20]. As LOX-1 is a scavenger receptor and STBEVs are, in essence, packages of lipoproteins, we propose that STBEVs contribute to endothelial dysfunction during pregnancy by activating the LOX-1 receptor. In the current study, we have investigated whether syncytiotrophoblast-derived particles such as STBEVs are able to induce endothelial dysfunction in pregnant rat uterine arteries and whether this is LOX-1 dependent. We hypothesized that STBEVs impair vascular function in pregnancy via activation of LOX-1 and increased superoxide production, which leads to decreased NO bioavailability.

## Methods

### Ethics approval

All animal experiments were conducted at the University of Alberta, Canada, and were approved by the University of Alberta Health Sciences Animal Policy and Welfare Committee in accordance with the Canadian Council on Animal Care Guidelines (AUP #242). The study protocol for human placentae was approved by the Oxfordshire Research Ethics Committee C and STBEV isolations were conducted in Prof. Ian Sargent’s laboratory at Oxford University, U.K. STBEVs were derived from the placenta according to their standard methods described in detail in the manuscript by Dragovic *et al*. [21]. In short, the placenta was collected from an uncomplicated nulliparous singleton pregnant woman directly after caesarian section (elective) (age 24; 39+1 weeks of gestation; BP 120/80 mmHg; no urinary protein) and, within 20 minutes, an intact lobule was perfused for 3h. The maternal perfusate was collected and centrifuged at high speed (150,000 g) to collect the STBEVs. To confirm the placental origin and normal size distribution of the STBEVs, flow cytometry and Nanoparticle Tracking Analysis were used as described previously [21]. Pellets were diluted in PBS to 1 mg protein ml^-1^ and frozen until their use in experiments. Written informed consent was obtained.

### Animals and experimental design

Three-month-old female Sprague Dawley rats were housed under a standard day:night cycle (10:14 hours) with *ad libitum* access to food and water. The presence of sperm in a vaginal smear following overnight mating with a male rat was designated as gestational day 0 of pregnancy. On gestational day 20, rats were sacrificed by exsanguination under inhaled isoflurane anesthesia. Main branch uterine arteries were isolated and cut into 2 mm pieces without side branches. Multiple 2 mm uterine artery segments were incubated for 24 hours at 4°C (as adapted from similar experiments published by others [22]) in each of the following groups: 1) physiologic salt solution (PSS) as a control, 2) PSS with LOX-1 blocking antibodies (TS20, 10 μg ml^-1^), 3) STBEVs (200 μg ml^-1^ in PSS), or 4) STBEVs (200 μg ml^-1^ in PSS) together with LOX-1 blocking antibodies (TS20, 10 μg ml^-1^). The STBEV concentration was based on previous studies [7]. There was no visible difference between the uterine artery segments; therefore each segment was randomly assigned to one of the experimental groups. Two of the incubated segments from each group were then used to assess arterial function using wire myography while the remaining segments (one to two segments per group) were snap frozen for subsequent analyses. LOX-1 blocking antibodies (TS20) were developed by Prof. Sawamura’s laboratory.

### Wire myography protocols

After 24 hours of incubation, segments of uterine artery were mounted on a wire myograph (DMT, Copenhagen, Denmark). Arteries were twice exposed to phenylephrine (10 μmol L^-1^, Sigma-Aldrich; with washout between doses) and once to methylcholine (MCh) (3 μmol L^-1^, Sigma-Aldrich) following the second phenylephrine dose, to ensure intact endothelial and smooth muscle function. To assess the NO contribution to vasodilation, arteries from each experimental group were pre-incubated for 30 minutes with or without N-nitro-l-arginine methyl ester hydrochloride (n = 12; L-NAME, 100 μmol L^-1^, Sigma-Aldrich). To assess the influence of superoxide production on vascular function, control and STBEV incubated arteries were pre-incubated for 30 minutes with or without superoxide dismutase (n = 8; polyethylene glycol SOD, 50 U mL^-1^, Sigma-Aldrich). Following incubation, arteries were pre-constricted with phenylephrine (3 μmol L^-1^) and vasodilator responses to MCh (0.1 nmol L^-1^ to 100 μmol L^-1^) were measured. Finally, to investigate endothelium-independent vasodilator function, arteries were pre-constricted with phenylephrine (3 μmol L^-1^) and responses to the exogenous NO donor sodium nitroprusside (n = 8; SNP, 0.1 nmol L^-1^ to 10 μmol L^-1^, Sigma-Aldrich) were assessed.

### Superoxide detection

Frozen sections of uterine artery (n = 8) were cut into 9 μm sections and stained for the presence of superoxide using dihydroethidium (DHE, Biotum, Inc. Hayward, CA, USA) staining. DHE reacts with superoxide to produce ethidium, which generates a red fluorescence that can be quantified. In short, arterial sections were thawed to room temperature for one minute and washed three times with Hanks’ Balanced Salt Solution (HBSS, Life Technologies, Burlington, ON, Canada) for 2 minutes each. Sections were then incubated with HBSS for 10 minutes at 37°C; which was then removed and diluted DHE solution (4 μmol L^-1^) was added for 30 minutes at 37°C. Afterwards, sections were washed thrice with HBSS (2 minutes each), covered with a coverslip, and fluorescent images were taken immediately.

### Endothelial nitric oxide synthase expression, nitrotyrosine levels and LOX-1 expression

Endothelial nitric oxide synthase (eNOS) expression, nitrotyrosine levels and LOX-1 expression in frozen uterine artery sections (9 μm) were measured using immunofluorescent staining. In short, sections were fixed in ice-cold acetone (-20°C) for 10 minutes and allowed to dry for another 10 minutes. Sections were washed 3 times for 5 minutes with phosphate buffered salt solution (PBS, pH 7.4) and incubated with blocking solution (2% BSA in PBS) for 60 minutes at room temperature. Subsequently, the blocking solution was aspirated and sections were incubated with anti-eNOS antibodies (NOS3, rabbit-anti-rat, 1:200, Santa Cruz Biotechnologies), anti-nitrotyrosine antibodies (rabbit-anti-nitrotyrosine, 1:50, Life Technologies) or anti-LOX-1 antibodies (rabbit-anti-rat, 1:50, Santa Cruz Biotechnologies) in 2% BSA in PBS overnight at 4°C. The next day, sections were washed with PBS 3 times for 5 minutes and incubated with secondary goat-anti-rabbit Alexa Fluor 546 (Cy3 wavelength) labeled antibodies (1:250; Molecular Probes/Thermo Fisher Scientific) in 2% BSA in PBS for 60 minutes at room temperature in the dark. Sections were then washed with PBS 3 times for 5 minutes, mounting medium with DAPI (nuclear staining, Vector Laboratories) was added and sections were covered and allowed to dry. Images were taken on the following day.

### Image analysis

Images of DHE, eNOS, nitrotyrosine and LOX-1 stained sections of uterine artery were taken using an Olympus IX81 fluorescence microscope with cellSens Dimensions software (Olympus). DHE eNOS, nitrotyrosine and LOX-1 mean staining intensity of the whole vessel was analyzed using ImageJ software. When two arterial segments were present in a sample, an average of the two mean intensities was taken.

### Statistical analyses

Statistical analyses were performed using GraphPad Prism software 6.0f (GraphPad software Inc., La Jolla, CA, U.S.). All data were tested for normality using the Shapiro-Wilk normality test. Myography data were summarized as percent maximal vasodilation or area under curve (AUC) and presented as mean ± standard error of the mean. Statistical analysis was performed for comparisons between control arteries and arteries exposed to STBEVs in the absence or presence of the LOX-1 blocking antibody or pegSOD using a two-way ANOVA with Bonferroni multiple comparisons post hoc test. The contribution of NO to endothelial vasodilation was quantified by calculating the delta change in AUC between arteries exposed to L-NAME and the controls and compared between the groups using a one-way ANOVA and Dunnet’s post hoc test. Comparisons of DHE staining between control arteries and arteries exposed to STBEVs or STBEVs + LOX-1 blocking antibody were analyzed using a nonparametric Kruskal-Wallis test with Dunn’s post hoc analysis. Comparisons of vascular responses to SNP and eNOS expression between control arteries and arteries exposed to STBEVs or STBEVs + LOX-1 blocking antibody were analyzed using a one-way ANOVA with Dunnet’s post hoc test. For all statistical tests, differences were considered significant if p<0.05.

### Materials

All drugs used for myography protocols were purchased from Sigma-Aldrich (St. Louis, MO, USA). The STBEVs were collected in Prof. Ian Sargent’s laboratory and were derived according to their standard methods. The LOX-1 blocking antibodies (TS20) were developed and supplied by Prof. Sawamura’s laboratory. DHE was purchased at Biotum, Inc. Hayward (CA, USA). HBSS was purchased from Life Technologies (Burlington, ON, Canada). The eNOS antibodies were obtained from Santa Cruz Biotechnologies, and the secondary goat-anti-rabbit Alexa Fluor 546-labeled antibodies from Molecular Probes/Thermo Fisher Scientific (Burlington, ON, Canada).

## Results

### STBEVs impaired MCh-mediated vasodilation in uterine arteries

**Fig 1:**
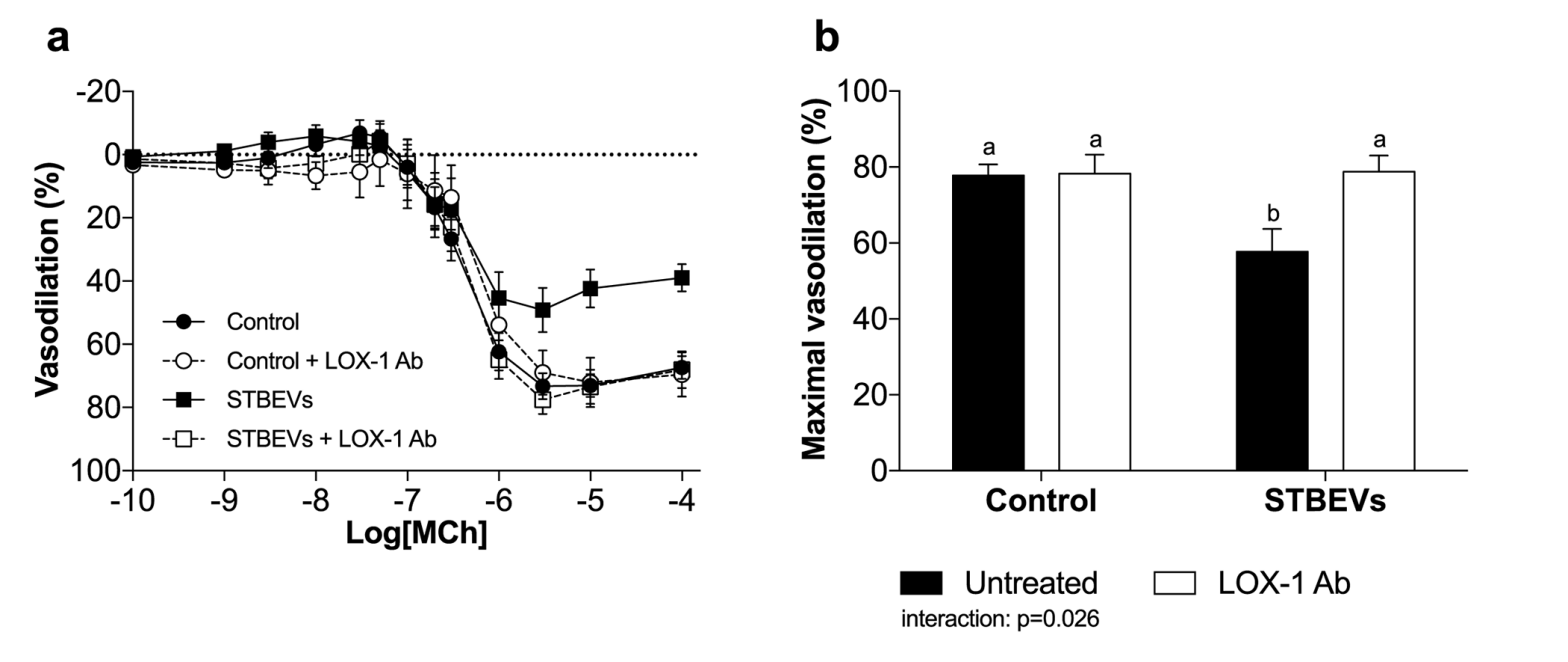
The effect of STBEV incubation on vascular responses to methylcholine. (A) Arteries exposed to STBEVs (solid squares, solid line) exhibited impaired vasodilation compared with control arteries (solid circles, solid line). Inhibition of LOX-1 restored endothelial responses in STBEV-exposed arteries (open squares, dashed line) to a level comparable with controls. LOX-1 inhibition had no effect in control arteries (open circles, dashed line). (B) Summary of myography data as percent maximal vasodilation. Data are presented as means ± SEM; two-way ANOVA, ‘a’ denotes statistical difference from ‘b’, p<0.05; n = 11–18/group.

Maximal MCh-induced vasodilation was reduced in STBEV-incubated uterine arteries (Fig 1A and 1B). The addition of LOX-1 blocking antibodies to arteries exposed to STBEVs resulted in an increased responsiveness to MCh back to responses comparable with controls (Fig 1A and 1B). The LOX-1 blocking antibodies had no effect on vasodilation in control vessels (Fig 1A and 1B).

### NO contribution to vasodilation was reduced in STBEV-incubated uterine arteries

**Fig 2:**
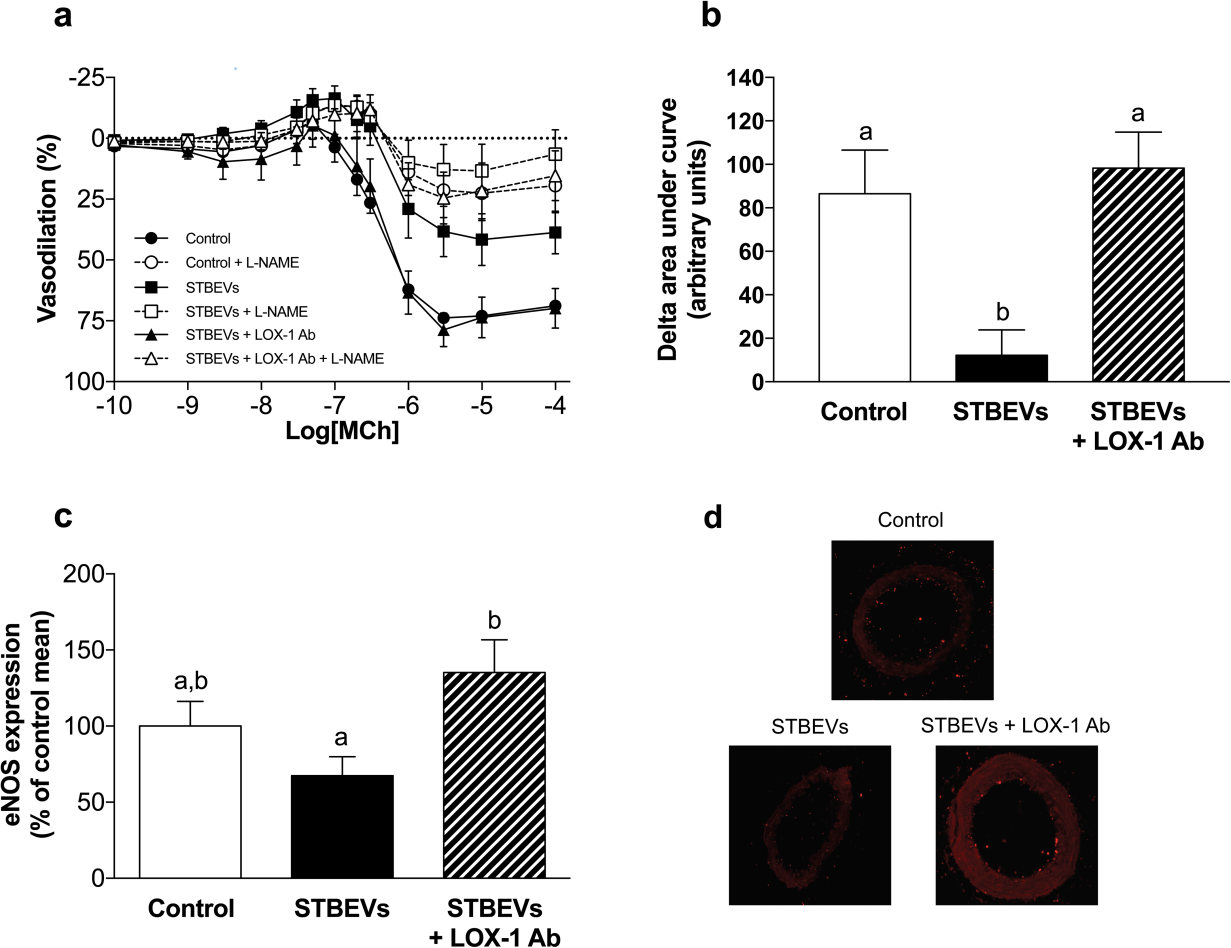
Changes in nitric oxide contribution to vasodilation after STBEV incubation. (A) Arteries exposed to STBEVs exhibited impaired vasodilation to methylcholine that was associated with reduced NO bioavailability (squares). Inhibition of LOX-1 improved NOS-mediated vascular responses in STBEV-exposed arteries (triangles) to a level comparable with controls (circles). (B) Quantitative summary of the NO contribution to endothelial vasodilation assessed as delta area under curve (arbitrary units). (C) Endothelial nitric oxide synthase (eNOS) expression in uterine arteries after 24-hour exposure in the absence (control) or presence of STBEVs, with or without a LOX-1 blocking antibody. The expression of eNOS was not significantly altered by exposure to STBEVs (solid bar) as compared with controls (open bars). Inhibition of LOX-1 in STBEV-treated arteries significantly increased eNOS expression (dashed bar). (D) Representative images of eNOS staining. Data are presented as means ± SEM; one-way ANOVA, ‘a’ denotes statistical difference from ‘b’, p<0.05; n = 6–12/group.

Inhibition of nitric oxide synthase (NOS) by L-NAME reduced maximal vasodilation to MCh in both control and STBEV-exposed (in the absence or presence of LOX-1 blocking antibodies) vessels (Fig 2A and 2B). The contribution of NO to vasodilation, as assessed by delta AUC, was decreased in STBEV-incubated vessels compared with controls; while incubation with the LOX-1 blocking antibody increased the NO contribution to vasodilation in STBEV-incubated vessels (Fig 2B). In addition to the production of superoxide [14], LOX-1 activation has also been shown to decrease eNOS expression [17, 18]. Compared to controls, exposure to STBEVs did not significantly alter eNOS expression; however, the inhibition of LOX-1 increased eNOS expression in STBEV-exposed arteries (Fig 2C and 2D). The LOX-1 blocking antibody did not alter eNOS expression in control arteries (mean ± SEM: 28.8 ± 11.2 a.u. control vs. 28.6 ± 7.4 a.u. control + LOX-1 antibody).

**Fig 3:**
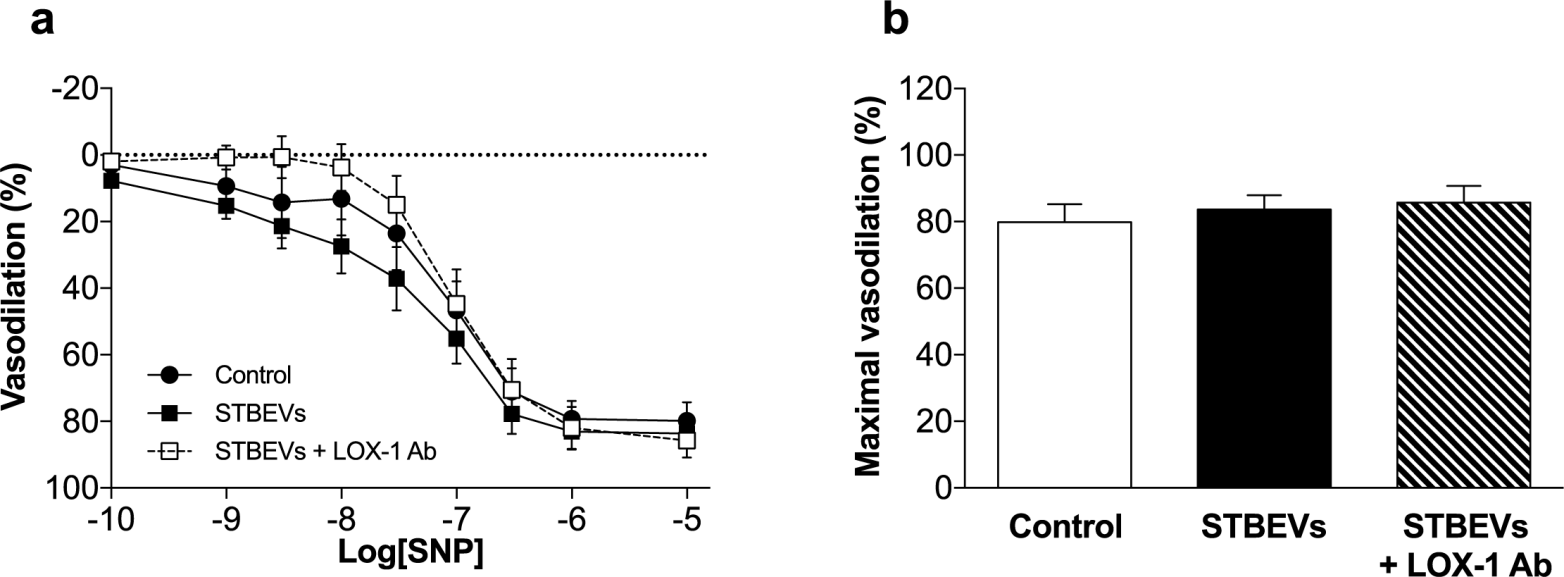
Intrinsic vascular smooth muscle cell function is not affected by STBEV incubation. (A) Vascular smooth muscle cell responses to the exogenous NO donor sodium nitroprusside (SNP) were not significantly different between controls (circles) or arteries exposed to STBEVs, with (open squares) or without (solid squares) a LOX-1 blocking antibody. (B) Summary of myography data as percent maximal vasodilation (E_max_). Data are presented as means ± SEM; one-way ANOVA; n = 6–8/group.

To distinguish whether the effects of STBEVs on NO-mediated vasodilation was the result of effects on altered endothelial or vascular smooth muscle function, we analyzed vascular responses to SNP, an exogenous NO donor. We found that vascular (smooth muscle) responses to SNP were not significantly different between control arteries and those exposed to STBEVs, or STBEVs in the presence of LOX-1 inhibition (Fig 3A and 3B).

### STBEV-induced impaired vasodilation is not mediated by superoxide anions

**Fig 4:**
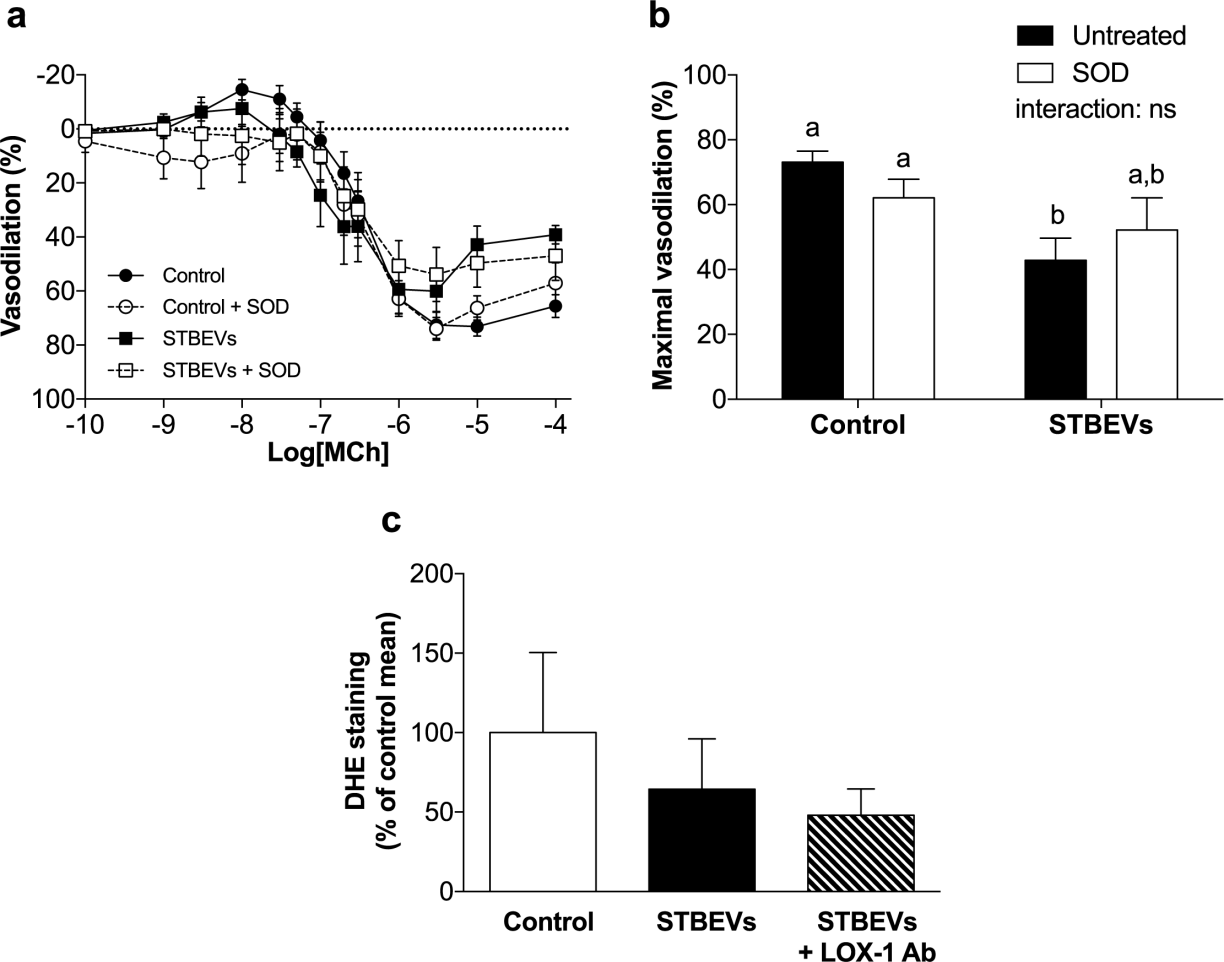
The effect of STBEVs on superoxide production in uterine arteries. (A) Vascular responses to methylcholine, an endothelium-dependent vasodilator, were impaired after overnight incubation with STBEVs (solid squares, solid line) as compared with controls (solid circles, solid line). However, scavenging of superoxide (pegSOD) did not significantly alter endothelial vasodilation in either control (open circles, dashed line) or STBEV exposed arteries (open squares, dashed line). (B) Summary of myography data as percent maximal vasodilation (E_max_). Data in A and B are presented as means ± SEM; two-way ANOVA, ‘a’ denotes statistical difference from ‘b’, ns = not significant; p<0.05. (C) Dihydroethidium (DHE) staining was not significantly different between control, STBEV, and STBEV + anti-LOX-1 antibody exposed arteries. Data in C are presented as median (range); Kruskal-Wallis test. n = 6–8/group.

LOX-1 receptor activation has been shown to increase superoxide production in diseased vasculature [12, 14]. Contrary to our hypothesis that STBEVs would activate LOX-1 and this would induce superoxide production, incubation with pegSOD did not improve MCh-induced vasodilation in STBEV-exposed arteries (Fig 4A and 4B). In addition, sections of uterine arteries were assessed for superoxide production using DHE staining and no differences were found between the groups (Fig 4C). This suggests that the STBEV-induced impairment in endothelial vasodilation was mediated via LOX-1 activation but not associated with increased superoxide production after 24 hours of exposure. Peroxynitrite is an oxidant formed by the reaction of superoxide with nitric oxide that affects vascular function [23]. Nitrotyrosine is one of the reaction products of peroxynitrite. Nitrotyrosine levels in uterine artery sections were not affected by STBEV stimulation (see Online Data Supplement, S1 Fig).

### LOX-1 expression after STBEV incubation in uterine arteries

LOX-1 staining was performed on uterine artery sections to check whether STBEV stimulation had any effect on LOX-1 expression. No changes were observed in LOX-1 expression between the experimental groups (see Online Data Supplement, S2 Fig).

All raw data is provided in S1 File.

## Discussion

In the current study, we have shown that STBEVs impaired endothelium-dependent vasodilation in uterine arteries, which appeared to be LOX-1 receptor mediated. We also demonstrated that arteries exposed to STBEVs exhibited an increased LOX-1 contribution to impaired NO-mediated vasodilation. However, vascular superoxide production was unaltered by exposure to STBEVs. Inhibition of LOX-1 in STBEV-incubated vessels increased eNOS expression. These data support our hypothesis that STBEVs can play a role in vascular dysfunction through the activation of LOX-1.

Our data have shown that STBEVs can increase LOX-1 mediated vascular activation and dysfunction. Endothelium-dependent vasodilation in uterine arteries was significantly inhibited by incubation with STBEVs and restored by the presence of LOX-1 blocking antibodies. To our knowledge, we are the first to show that this STBEV-induced endothelial impairment could potentially be mediated via the LOX-1 receptor. LOX-1 activation has previously been suggested to play a role in the endothelial dysfunction commonly seen in cardiovascular diseases, for example, atherosclerosis [19] and during pregnancy [10, 12]. Previous literature has examined the effects of STBEVs on vascular function, but has resulted in conflicting reports. In line with our findings, Cockell *et al*. have shown that STBEVs impair endothelial-dependent vasodilation in human omental fat arteries [7]. In contrast, while using a similar vascular bed as the current study, Van Wijk *et al*. did not find any effect of STBEVs in human myometrial arteries [8]. However, this difference could be attributed either to their use of lower STBEV concentrations (i.e. not sufficient to activate LOX-1) or the use of vessels from a different species. By utilizing a pregnant rat model, we obtained uterine arteries for our experiments because they constitute the most important vascular bed supplying the growth and development of the uterus, placenta and fetus during pregnancy.

LOX-1 associated impairment of vasodilation after STBEV stimulation may have several intracellular causes. We have shown that the overall endothelial NO contribution to vasodilation was lower in STBEV incubated arteries as compared with controls. In addition, no change in SNP-induced vasodilation was observed after STBEV stimulation; therefore, smooth muscle cell responses to NO appear unchanged and the STBEV-induced effects appear to be endothelium specific. Activation of LOX-1 by oxLDL has been shown to induce activation of Rho kinase A which may liberate arginase from the mitochondria and increase catabolism of L-arginine, the primary substrate for NOS [24]. In addition, oxLDL has also been shown to decrease eNOS activity in bovine aortic endothelial cells by dephosphorylation, a process that was LOX-1 mediated [25]. Hence, the impaired NO contribution to vasodilation by STBEVs could be mediated via 1) a reduction in NO production by NOS as a result of a LOX-1-induced increase in arginase production and decreased L-arginine availability; or 2) a lack of NO production due to changes in eNOS phosphorylation/activation [26] and/or intracellular localization [27], aspects which could be investigated in further studies.

While previous studies have shown that LOX-1 activation can decrease eNOS expression [17, 18, 28], and our functional data demonstrated that LOX-1 activation by STBEVs reduced NO-mediated relaxation, no changes in vascular eNOS expression were observed after STBEV exposure. Our eNOS measurements included both endothelial and smooth muscle cells. It has been previously demonstrated that all three NOS isoforms are strongly expressed in smooth muscle cells of various types of blood vessels, and specifically in small arterioles [29]. In addition, eNOS has been shown to be expressed in uterine artery vascular smooth muscle cells [30]. Interestingly, there appears to be a regulatory relationship between LOX-1 and eNOS expression as we found that blocking the LOX-1 receptor increased vascular eNOS expression in STBEV-stimulated vessels, while LOX-1 blocking on its own had no effect. In line with these findings, other investigators have shown that eNOS expression can be decreased by LOX-1 activation and returned to control levels after blocking the LOX-1 receptor [17, 18, 28].

LOX-1 activation induces NADPH oxidase dependent superoxide production in bovine aortic and human umbilical vein endothelial cells [14, 31, 32]. Superoxide is then able to scavenge NO, thus reducing NO bioavailability and leading to peroxynitrite formation [14, 31, 32], both of which result in impaired vascular function. In an *ex vivo* setting, oxLDL-induced impairment of NO mediated vasodilation of mice coronary arterioles was also shown to be LOX-1 and NAPDH oxidase dependent [33]. Interestingly, even though LOX-1 activation was apparent, we did not observe increased superoxide or peroxynitrite (nitrotyrosine) production in sections of STBEV-incubated rat uterine arteries; nor did we observe an improvement of vascular function after adding pegSOD to STBEV-exposed vessels. However, as reactive oxygen species, including superoxide, are short-lived [34], this finding might not have captured an STBEV-induced production of superoxide at an earlier phase of stimulation. Hence, it is possible that superoxide or peroxynitrite may be produced at earlier stages, which activates other signaling pathways that continue to influence vasodilation after 24 hours.

A diverse range of LOX-1 receptor ligands have been described [35] and, because STBEVs are essentially packages of lipoproteins, we proposed that the LOX-1 scavenger receptor might also be activated by STBEVs. In the current study we are, to our knowledge, the first to show that STBEVs could indeed be LOX-1 ligands. Since this study was conducted as an initial proof-of- principle investigation to determine whether syncytiotrophoblast derived particles *per se* could activate LOX-1, we used a single STBEV-sample that contained a heterogeneous population of extracellular vesicles. The biological relevance of the presented data remains to be further investigated, however, our data suggest that STBEVs are indeed able to activate LOX-1. Interestingly, women with the preeclampsia present with vascular endothelial dysfunction while at the same time this pregnancy disorder has been associated with increased STBEV concentrations [3, 36] in combination with higher LOX-1 expression [12]. Together with our findings, this could suggest that STBEV-induced LOX-1 mediated vascular dysfunction could potentially play a role in this syndrome. This would be of interest in future studies using STBEVs derived from pregnancies complicated by preeclampsia.

During pregnancy, the maternal vasculature is constantly exposed to circulating STBEVs over several months of gestation. The concentrations of STBEVs measured in plasma from pregnant (and preeclamptic) women (20–100 ng ml^-1^) [36–38] are lower than the STBEV concentration of 200 μg ml^-1^ that we utilized in our current experiments. This higher concentration of STBEVs for a shorter (24 hours) duration of exposure was used to enable us to assess the possible role of LOX-1 in an *ex vivo* bioassay and is in-line with other previous studies [7]. We used overnight incubation (similar to the study by van Wijk *et al*. [22]) to ensure there was enough time for potential interaction, and we have not observed any obvious differences in basal vascular function between overnight-incubated and freshly isolated arteries.

As STBEVs are heterogeneous [1], it may be speculated that mainly vesicles that contain oxidized lipids are able to bind LOX-1. In addition, it has been shown that phosphatidylserine can bind and activate the LOX-1 receptor [39], and these lipoproteins have also been shown to be present on STBEVs [40]. Whether STBEVs are internalized by endocytosis upon LOX-1 activation and have subsequent intracellular effects, which is a general feature of scavenger receptors [35], remains to be studied. Scavenger receptors play an important role in distinguishing self from non-self and in the inflammatory response [35]. Thus it is tempting to speculate from our results that activation of monocytes and peripheral blood mononuclear cells by STBEVs [2, 5] could potentially also be LOX-1 receptor (or other scavenger receptor) dependent. This remains to be investigated further but would greatly enhance the therapeutic potential of LOX-1 blocking.

From a clinical perspective, endothelial dysfunction is a key point of convergence underlying many pathologies; however, the exact mechanism of how placental circulating factors affect the maternal vasculature is still under investigation. In this study, we have provided evidence that STBEVs play a role in the vascular dysfunction. STBEVs, containing vesicles and exosomes derived from placental syncytiotrophoblasts, impaired endothelial vasodilation and were associated with reduced NO bioavailability via the LOX-1 receptor. Not only does this increase our collective understanding of the vascular pathophysiology, but it also provides insight into potential therapeutic strategies by targeting the LOX-1 pathway.

## Supporting information

S1 Fig The effect of STBEVs on nitrotyrosine levels in uterine arteries. No differences in nitrotyrosine levels were observed between all of the experimental groups. Bars represent means ± SEM; two-way ANOVA. ns = not significant. n = 6–7/group.
(PDF)

S2 Fig LOX-1 expression in uterine arteries after incubation with STBEVs. No differences in uterine artery LOX-1 expression were found between the experimental groups. Bars represent means ± SEM; two-way ANOVA. ns = not significant. n = 6–7/group.
(PDF)

S1 File Raw data files. (ZIP)
