# Supplementary material for: Syncytiotrophoblast extracellular vesicles impair rat uterine vascular function via the lectin-like oxidized LDL receptor-1
Source: PLoS One. 2017 Jul 3;12(7):e0180364. doi: 10.1371/journal.pone.0180364 (PMC5495440; doi:10.1371/journal.pone.0180364)
Supplement: S1 Fig — No differences in nitrotyrosine levels were observed between all of the experimental groups. Bars represent means ± SEM; two-way ANOVA. ns = not significant. n = 6–7/group. (PDF) [file pone.0180364.s001.pdf]

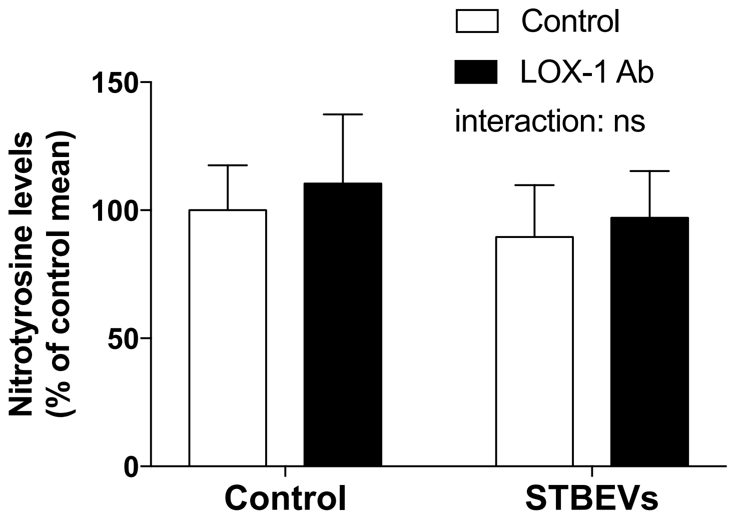

S1 Fig. The effect of STBEVs on nitrotyrosine levels in uterine arteries. No differences in nitrotyrosine levels were observed between all of the experimental groups. Bars represent means ± SEM; two-way ANOVA. ns=not significant. n=6-7/group.
