# Supplementary material for: Syncytiotrophoblast extracellular vesicles impair rat uterine vascular function via the lectin-like oxidized LDL receptor-1
Source: PLoS One. 2017 Jul 3;12(7):e0180364. doi: 10.1371/journal.pone.0180364 (PMC5495440; doi:10.1371/journal.pone.0180364)
Supplement: S2 Fig — No differences in uterine artery LOX-1 expression were found between the experimental groups. Bars represent means ± SEM; two-way ANOVA. ns = not significant. n = 6–7/group. (PDF) [file pone.0180364.s002.pdf]

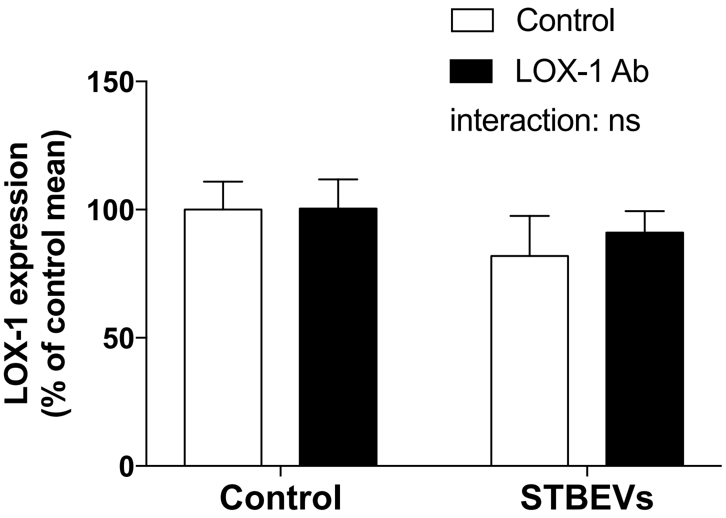

S2 Fig. LOX-1 expression in uterine arteries after incubation with STBEVs. No differences in uterine artery LOX-1 expression were found between the experimental groups. Bars represent means ± SEM; two-way ANOVA. ns=not significant. n=6-7/group.
